# A Case Report of a Patient With Prostate Adenocarcinoma Metastatic to the Posterior Peritoneum Despite the Negative Preoperative Prostate-Specific Membrane Antigen (PSMA) Positron Emission Tomography/Computed Tomography (PET/CT) Scan

**DOI:** 10.7759/cureus.39948

**Published:** 2023-06-04

**Authors:** Celeste Shoeleh, John Graff, Trushar Patel

**Affiliations:** 1 Department of Urology, University of South Florida, Tampa, USA; 2 Department of Pediatric Oncology, USF (University of South Florida) Health, Tampa, USA

**Keywords:** urology, prostate-specific antigen (psa), prostate-specific membrane antigen, imaging, metastatic prostate carcinoma

## Abstract

Despite the role of prostate-specific antigen (PSA) screening and the multitude of therapies available, prostate cancer (PCa) remains a leading cause of cancer-related morbidity and mortality. For many patients diagnosed with PCa, clinical and radiographic staging are critical components for management decisions. PCa staging with the use of imaging modalities such as MRI and bone scintigraphy is recommended in patients with newly diagnosed intermediate or high-risk PCa and in patients with biochemical recurrence; it is also recommended for monitoring the patient’s response to treatment for diagnosed PCa.

Prostate-specific membrane antigen (PSMA) positron emission tomography/computed tomography (PET/CT), recently approved in 2021, is an imaging modality that has been shown to have a greater sensitivity, specificity, and negative likelihood ratio than conventional imaging modalities such as CT, bone scintigraphy, and MRI in prostate cancer staging. Despite the improvement in staging that PSMA-PET/CT can provide, our current report details a false-negative result in detecting a rare PCa metastasis to the peritoneum, which was found at the time of an attempted radical prostatectomy. Although the patient had a negative preoperative PSMA-PET/CT and was presumed to be non-metastatic, the prostatectomy was aborted because the patient was unexpectedly found to have peritoneal metastasis.

## Introduction

Prostate cancer (PCa) is the second leading cause of cancer death in males in the United States and can have a poor prognosis if metastasis outside of regional lymph nodes is detected [1]. The current gold standard for PCa screening is elevated prostate-specific antigen (PSA); diagnosis is confirmed via prostate needle biopsy. Detecting PCa metastasis has not been standardized, and to date, multiple imaging modalities can be used in the initial detection and staging of PCa such as CT, bone scintigraphy, MRI, and the newly developed prostate-specific membrane antigen (PSMA)-positron emission tomography/computed tomography (PET/CT) [1-3]. The sensitivity and specificity ranges are 65.9%-71% and 95%-98.9% for PSMA-PET/CT, 7%-42% and 82%-100% for CT scan, 79% and 82% for bone scintigraphy, and 18.8%-69.7% and 78.6%-97.6% for MRI, respectively [3].

CT has been widely used to detect lymph nodes and distant metastasis for PCa. While CT alone can be used with limited efficacy, PET/CT is often utilized for better specificity and sensitivity [1]. Four PET/CT modalities are available such as choline-PET/CT, fluorodeoxyglucose (FDG)-PET/CT, sodium fluoride (NaF) PET/CT, and PSMA-PET/CT. Choline-PET/CT is based on the tumor’s ability to utilize choline, and FDG PET/CT is based on the tumor’s fluorodeoxyglucose uptake. However, these imaging modalities are not the standard practices in the detection of PCa due to their limited sensitivities for smaller metastases and broad ranges. Choline-PET/CT was reported to have a sensitivity ranging from 73%-91%, and FDG PET/CT had a broad range of sensitivity of 4.0%-80% [1,4]. NaF-PET/CT detects bone metastasis and has shown to have significantly better sensitivity and specificity than conventional bone scintigraphy [3,5].

PSMA-PET/CT detects PCa cells that overexpress PSMA. Although PSMA-PET/CT is a recent advancement, it has been shown to have a higher sensitivity and a negative likelihood ratio in the initial diagnosis and staging of PCa than other modalities [3,6,7]. However, as detailed in this report, PSMA-PET/CT may not be useful in the staging of PCa if patients have rare metastasis and low clinical suspicion of advanced disease.

In this case report, we describe a 75-year-old male with PCa who was expected to undergo a robotic radical prostatectomy after PSMA-PET/CT was negative for distant metastasis. However, the surgery was aborted due to advanced peritoneal metastatic disease confirmed via intraoperative frozen section.

## Case presentation

The patient is a 75-year-old male who initially presented with elevated PSA levels of 18 and 21 ng/ml. A multiparametric prostate MRI was performed, which revealed one PI-RADS 5 (Prostate Imaging Reporting and Data System category 5) lesion near the left base of the prostate and a second PI-RADS 4 lesion at the left peripheral zone. The prostate was measured to be 55 ccs. No evidence of lymphadenopathy or extracapsular disease was identified on MRI.

**Figure 1:**
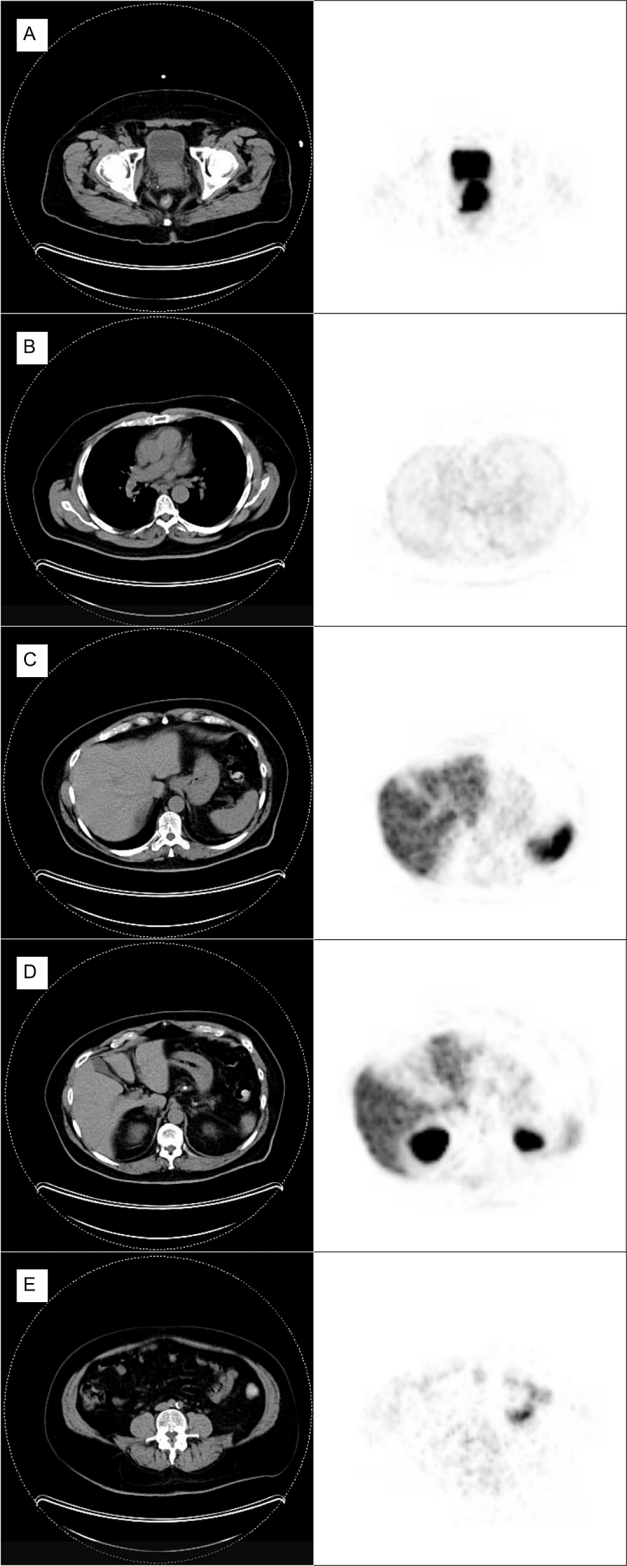
PSMA-PET/CT representative images (A) Prostate shows an abnormal expression of PSMA consistent with PCa. No evidence of pelvic lymph node metastasis was found. (B) Mediastinum shows no abnormal expression of PSMA and no evidence of pulmonary lymph node metastasis. (C) The liver shows no abnormal expression of PSMA. Imaging is consistent with the liver’s typical metabolic activity. (D) Adrenal glands show increased metabolic activity consistent with their typical activity. The patient also has nephrolithiasis, which may have contributed to the imaging observed. (E) Retroperitoneal lymph nodes do not show evidence of increased metabolic activity. PSMA: Prostate-specific membrane antigen; PET/CT: Positron emission tomography/computed tomography; PCa: Prostate cancer.

A transrectal MRI-fusion prostate biopsy was then performed, obtaining 14 total cores. This demonstrated Gleason 5+4=9 disease in seven out of 14 cores. A digital rectal exam (DRE) revealed a symmetrically enlarged and firm, but not fixed, prostate. Based on the clinical findings, the patient underwent further staging with PSMA-PET/CT post-biopsy, which was negative for metastatic disease, using 11.0 mCi (F-18) Pylarify® as a radiotracer (Figure 1).

**Figure 2:**
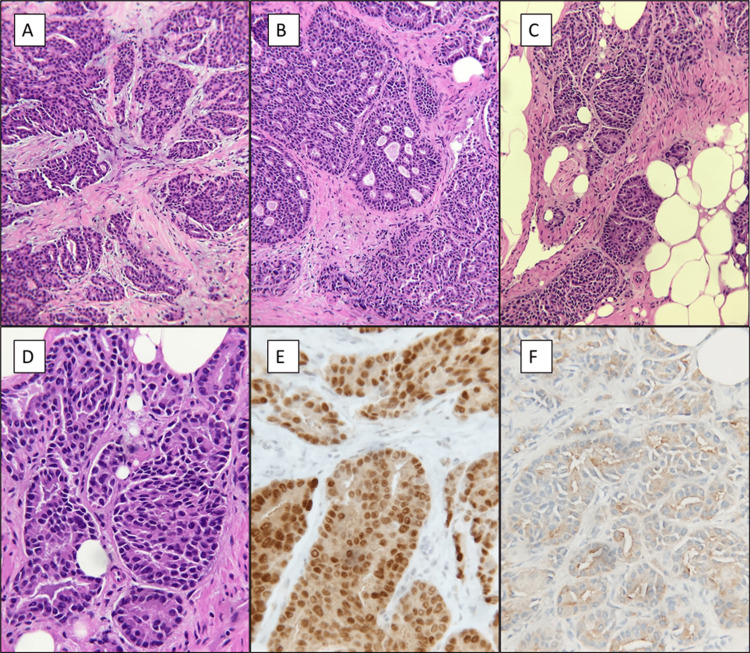
Sample images of intraoperative peritoneal biopsy demonstrating metastatic prostate adenocarcinoma (A-C) Low-power magnification, (D) high-power magnification, (E) NKX3.1 immunostain, and (F) PSA immunostain. PSA: Prostate-specific antigen.

Based on these data, the patient was counseled on treatment options and elected to proceed with a robot-assisted laparoscopic radical prostatectomy. After obtaining laparoscopic access and robot docking, when the pelvis was evaluated, an indurated mass was found on the peritoneal surface of the posterior bladder at the level of the cul-de-sac. A frozen section biopsy confirmed the diagnosis of metastatic PCa (Figure 2). On further intraoperative evaluation and DRE, we deemed the prostate to be completely fixed to the bladder. After discussion with the family, the procedure was aborted as we felt that a complete resection would not be possible without a potential pelvic exenteration. The patient was counseled on remaining treatment options including orchiectomy, androgen deprivation therapy, and radiation therapy.

**Figure 3:**
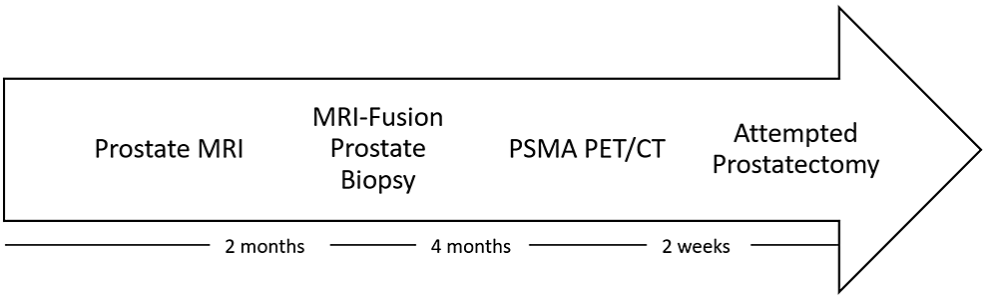
Timeline of events This figure shows the timeline between the procedures. PSMA: Prostate-specific membrane antigen; PET/CT: Positron emission tomography/computed tomography.

A summary of events is included in Figure 3.

## Discussion

In PCa, the most common metastatic sites include bone, distant lymph nodes, the liver, and the thorax. In contrast, rarer sites include the brain, digestive system, retroperitoneum, and adrenal glands [8,9]. Multiple imaging modalities including PSMA-PET/CT, bone scintigraphy, CT, MRI, and NaF PET/CT are available to be used in the detection and staging of PCa [3,5].

Currently, PSMA-PET/CT has been shown to have greater sensitivity, specificity, and negative likelihood ratio in detecting lymph nodes and distant metastasis compared to conventional imaging like CT and MRI [3-7]. Recent studies also suggest that PSMA-PET/CT may have greater sensitivity in detecting bone metastasis when compared to traditional bone scintigraphy [7,10]. However, there are limited studies on the PSMA-PET/CT’s ability to detect extraprostatic disease in sites such as the peritoneum as peritoneal metastasis is rare [11]. PSMA-PET/CT was successful in detecting peritoneal metastasis when clinicians had already suspected distant metastases due to steeply increased PSA values, low testosterone levels, and concerning CT findings as per the few case reports that detail peritoneal metastases detected via PSMA-PET/CT [12,13]. In those reports, PSMA-PET/CT was not used to rule out disease progression in a suspected stage T1 PCa (cancer limited to the prostate), as in our report, but rather to confirm the clinical suspicion.

In addition, one case report found that pre-therapeutic PSMA-PET/CT was negative for extraprostatic lesions for locally advanced prostate cancer until one year later when PSA values drastically increased and an updated PSMA-PET/CT revealed diffuse metastases [14]. These cases indicate that PSMA-PET/CT may only be useful in detecting distant and rare metastases when clinical suspicion is already present and may not be suitable for ruling out advanced PCa. Although PSMA-PET/CT may be useful in detecting metastasis to lymph nodes and bone, its sensitivity and negative likelihood ratio in detecting rarer metastasis are still in question and calls for further research.

## Conclusions

This study reports the diagnosis of Stage IVB, M1 metastatic adenocarcinoma of prostatic origin to the peritoneum despite a negative preoperative PSMA-PET/CT result. Although PSMA-PET/CT has been shown to have better sensitivity, specificity, and negative likelihood ratio than conventional imaging modalities, it may not be useful in ruling out such rare PCa metastasis before radical prostatectomy.
